# The Role of Fucoxanthin in Non-Alcoholic Fatty Liver Disease

**DOI:** 10.3390/ijms24098203

**Published:** 2023-05-03

**Authors:** Jessica Winarto, Dae-Geun Song, Cheol-Ho Pan

**Affiliations:** 1 Division of Bio-Medical Science and Technology, KIST School, University of Science and Technology, Seoul 02792, Republic of Korea; jessicawinarto@kist.re.kr (J.W.); dsong82@kist.re.kr (D.-G.S.); 2 Natural Product Informatics Research Center, KIST Gangneung Institute of Natural Products, Gangneung 25451, Republic of Korea; 3 Microalgae Ask US Co., Ltd., Gangneung 25441, Republic of Korea

**Keywords:** liver, non-alcoholic, obesity, thermogenesis, fucoxanthin, lipid, fibrosis

## Abstract

Chronic liver disease (CLD) has emerged as a leading cause of human deaths. It caused 1.32 million deaths in 2017, which affected men more than women by a two-to-one ratio. There are various causes of CLD, including obesity, excessive alcohol consumption, and viral infection. Among them, non-alcoholic fatty liver disease (NAFLD), one of obesity-induced liver diseases, is the major cause, representing the cause of more than 50% of cases. Fucoxanthin, a carotenoid mainly found in brown seaweed, exhibits various biological activities against NAFLD. Its role in NAFLD appears in several mechanisms, such as inducing thermogenesis in mitochondrial homeostasis, altering lipid metabolism, and promoting anti-inflammatory and anti-oxidant activities. The corresponding altered signaling pathways are the β3-adorenarine receptor (β3Ad), proliferator-activated receptor gamma coactivator (PGC-1), adenosine monophosphate-activated protein kinase (AMPK), peroxisome proliferator-activated receptor (PPAR), sterol regulatory element binding protein (SREBP), nuclear factor kappa B (NF-κB), mitogen-activated protein kinase (MAPK), protein kinase B (AKT), SMAD2/3, and P13K/Akt pathways. Fucoxanthin also exhibits anti-fibrogenic activity that prevents non-alcoholic steatohepatitis (NASH) development.

## 1. Introduction

Chronic liver disease (CLD) is the leading disease resulting in death in the world. There were approximately 1.5 billion cases of CLD worldwide in 2020, with 1.32 million deaths in 2017, which affected men more than women by a two-to-one ratio [1,2]. Originally, viral hepatitis was the major etiology of CLD, but recently, obesity and alcohol consumption have become increasingly common factors for CLD. CLD caused by a viral infection can be treated with proper medication and prevented with vaccination. It can also be easily and precisely detected based on the tests for infection, unlike non-alcoholic liver disease [3]. Non-alcoholic fatty liver disease (NAFLD) occurs when fat accumulates excessively in the liver due to an obesogenic diet and lifestyle [4]; hence, diagnosing NAFLD is challenging. The metabolic dysregulation caused by obesity disrupts the body’s homeostasis and affects the function of the liver. Prevention and treatment of obesity are thus critically important to combatting and preventing NAFLD.

Fucoxanthin, a naturally derived carotenoid compound, has shown anti-obesity effects [5,6,7,8]. It was first isolated from brown algae and seaweed such as Fucus, Dictyota, and Laminaria by Willstätter and Page in 1914 [9]. Brown seaweeds are a common dietary food in many parts of Asia and are found naturally in open seas, where they are exposed to metals as well as metalloids [10]. It is now commercially produced by various technologies [11], such as bioreactors using microalgae and diatoms. As a natural compound, it is hypothesized to have less severe side effects with lower toxicity levels. Fucoxanthin shows various kind of bioactivities, including anti-obesity effects that can be used for the treatment and prevention of NAFLD. Here, in order to better understand fucoxanthin’s activity on NAFLD, we review the literature on the activity of fucoxanthin in NAFLD and its signaling pathways.

## 2. Structure and Metabolites of Fucoxanthin

Fucoxanthin is a carotenoid mainly found in brown seaweed, acting as a photosynthetic pigment along with chlorophyll a and c, and β-carotene [12]. With a molecular weight of 658.9 g/mol, fucoxanthin (C_42_H_58_O_6_) is located in the photosynthetic organ of brown seaweed and microalgae and is responsible for photochemical events [13,14]. It has a unique structure with a wide range of inherent activities. There is an allenic bond, a conjugated carbonyl, a 5,6-monoepoxide, and an acetyl group [15,16]. An allenic bond is a condition where one carbon atom has two double bonds with each adjacent carbon [17].

Due to the structure of fucoxanthin, it mainly has two biological functions: singlet oxygen quenching and free radical scavenging [18]. The singlet oxygen quenching activity is due to the carbon double bonds located in the backbone of fucoxanthin. It mainly depends on physical quenching without any chemical reaction [19]. It works by transferring the energy in the singlet oxygen molecules to the conjugated double bond in fucoxanthin [20,21]. The excited fucoxanthin can dissipate energy into the environment, returning it to a ground state [19]. That will bring the fucoxanthin molecule back to its original state. Singlet oxygen quenching activity depends on the number of conjugated carbon double bonds [22,23]. The higher the number of conjugated double bonds, the more energy that can be transferred from the singlet oxygen molecule to the carotenoid.

Meanwhile, the free radical scavenging ability of fucoxanthin is due to the functional groups in the terminal rings [19,24]. They function as electron acceptors and electron donors, as well as in adduct formation. Moreover, there is an allenic bond in fucoxanthin. Among the 700 carotenoids in nature, there are about 40 types of carotenoids, which have an allenic bond, including fucoxanthin [25]. The allenic bond provides carotenoids with a higher activity than alkenes and a peculiar axial chirality [26] that contributes to the activity of fucoxanthin.

**Figure 1:**
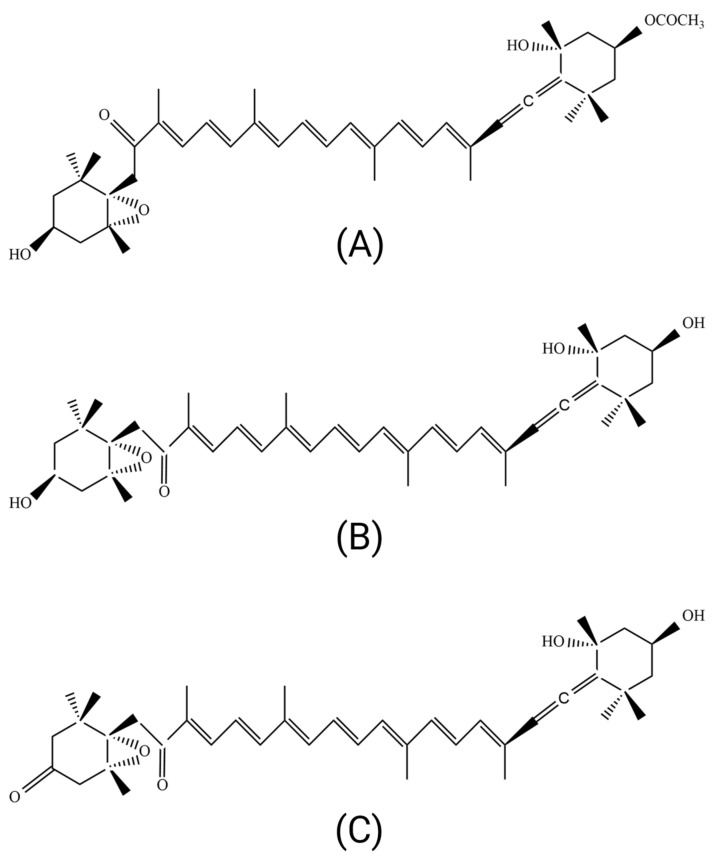
**Fucoxanthin and its metabolites.** (**A**) Fucoxanthin, (**B**) fucoxanthinol, and (**C**) amarouciaxanthin A.

Fucoxanthin can be absorbed into the human body through the digestive system at the intestinal level. Fucoxanthin is metabolized in the liver via fucoxanthinol to amarouciaxanthin A (Figure 1), requiring the cofactor nicotinamide adenine dinucleotide phosphate (NADP) [27,28,29]. Several enzymes are also involved in the gastrointestinal tract, such as lipase and cholesterol esterase. It was reported that the proportions of fucoxanthin, fucoxanthinol, and amarouciaxanthin A in the adipose tissue were 13%, 32%, and 55%, respectively, whereas in other tissues, including the liver, lungs, kidney, heart, and spleen, were 1–11%, 63–76%, and 20–26%, respectively [6,30]. Other than fucoxanthinol and amarouciaxanthin A, another fucoxanthin metabolite derived from fucoxanthinol, halocynthiaxanthin, has been isolated from *Undaria pinnatifida* [12]. This metabolite has not yet been fully studied, and discovering other metabolites of fucoxanthin is possible.

Fucoxanthin toxicity has been tested through several experiments, including in animals and humans. In ICR mice, fucoxanthin showed no mortality and no abnormalities in a single-dose study at 1000 and 2000 mg/kg, as well as 500 and 1000 mg/kg in a repeated-dose study for 30 days [14,31]. A single oral dose study was also performed on rats and showed no toxicity with a fucoxanthin administration of 200 mg/kg body weight [32]. It is also declared safe at 0.5% *w/v* for application on human skin [33]. Hence, the administration of fucoxanthin has been determined to be safe, and the physiological activity of fucoxanthin is considered to have great potential.

## 3. Role of Fucoxanthin in Non-Alcoholic Fatty Liver Disease

NAFLD occurs when the rate of hepatic fatty acid uptake is greater than its oxidation [34]. NAFLD is closely associated with obesity and the increase in intrahepatic triglyceride (IHTG) [35]. This occurs with an imbalance in food intake and energy expenditure, resulting in fat accumulation [36]. Triglycerides accumulate in the liver and disrupt the body’s metabolism, from the normal state to the hypercaloric state, when the energy produced inside the body is more than sufficient; hence, it is stored as a lipid [37]. Obesity increases mortality in NAFLD patients [38]. The rising number of NAFLD cases is closely related to the rising trend of obesity [39]. It is presumed that combating obesity is important for the treatment and prevention of NAFLD.

Fundamentally, there are two ways to overcome obesity: increasing energy expenditure or decreasing energy gain by controlling food intake [40]. Either way, the system works by adjusting the hypercaloric metabolic state of the body back to the homeostatic state. Fucoxanthin is known to have an anti-obesity effect, proven through different kinds of experiments, including cell culture, animal models, and human studies. The anti-obesity mechanisms of fucoxanthin are categorized into two classes: inducing thermogenic activity and altering lipid metabolism. Altering lipid metabolism is a strategy that can be effectively used against NAFLD [41].

**Figure 2:**
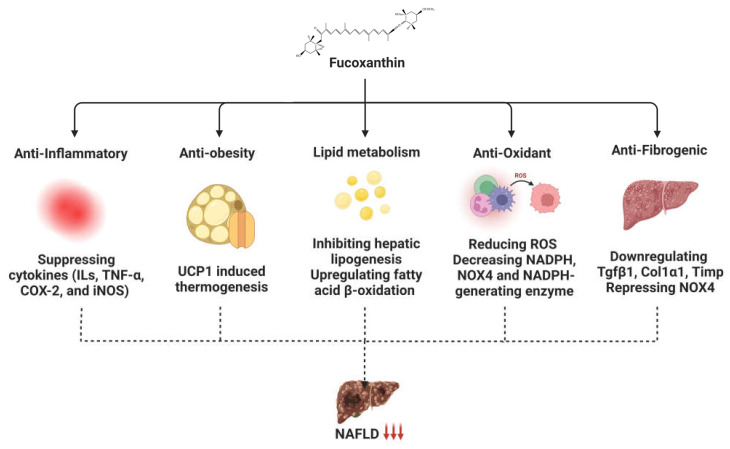
**The role of fucoxanthin against NAFLD.** Fucoxanthin exhibits anti-inflammatory, anti-obesity, anti-oxidant, and anti-fibrogenic activities and alters lipid metabolism in NAFLD.

Fucoxanthin has also been reported to reduce hepatic injury by decreasing hepatic fat accumulation and liver weight gain in a choline-deficient, L-amino-acid-defined high-fat diet (CDAHFD), non-alcoholic steatohepatitis (NASH) mouse model. It decreased hepatic lipid oxidation and NASH inflammation by inhibiting the production of chemokines [42]. It also alleviates lipid peroxidation in hepatocytes, resulting in the suppression of lipid accumulation [43]. Fucoxanthinol and amarouciaxanthin A have also been found to have an anti-inflammatory effect against NASH by down-regulating the hepatic stellate cell marker [42]. In conclusion, fucoxanthin is a promising therapeutic for inhibiting hepatic inflammation and preventing fibrosis in liver disease. The role of fucoxanthin in liver diseases, especially NAFLD, can be explained through multiple mechanisms, such as thermogenesis-induced anti-obesity activity, altered lipid metabolism, and anti-inflammatory, anti-oxidant, and anti-fibrogenic activities (Figure 2).

### 3.1. Fucoxanthin Affects Mitochondrial Homeostasis through Thermogenic Activity

Mitochondria play a major role in human health. Their role is centered on homeostasis and energy metabolism, including maintaining and producing the energy needed by the human body [4]. The disruption of mitochondrial homeostasis and elevated oxidative stress are commonly observed in fatty liver disease patients [44] and are characterized by a reduction in respiratory chain activity and impaired mitochondrial β-oxidation [45].

**Figure 3:**
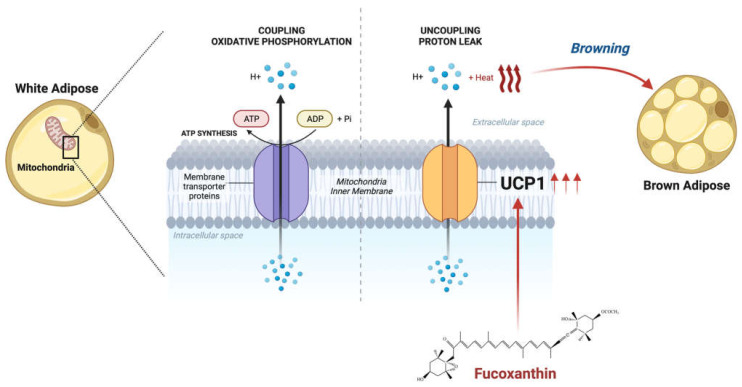
**Fucoxanthin-induced thermogenic activity.** Fucoxanthin induces the activation of UCP-1, leading to browning of white adipose tissues. Uncoupled proton leak will occur instead of coupling with oxidative phosphorylation along with heat release (adapted from Huang, (2022)) [50].

Many biological activities are performed inside mitochondria. One of them is oxidative phosphorylation. Mammalian cells synthesize energy through oxidizing substrates in inside mitochondria. One of the chemical reactions involved is oxidative phosphorylation located in mitochondria. During oxidative phosphorylation, free energy is converted into the displacement of adenosine triphosphate (ATP) in an equilibrium reaction. However, with uncoupling protein, hydrogen and heat are released instead of ATP synthesis through the uncoupling to ATP synthase, bypassing ATP synthase, and is hence called an uncoupling protein [46,47,48], as shown in Figure 3. Proton leak (hydrogen leak) in mitochondria is expected to occur through electron escape from mitochondrial oxidoreductase to generate superoxide [49].

Fucoxanthin exhibits anti-obesity effects, mainly through thermogenic effects via mitochondrial uncoupling protein 1 (UCP1) [5,8], as shown in Figure 3. UCP1, a 32 kDa protein, is an inner mitochondrial membrane protein that is a molecular basis for the protonophore activity in the mitochondrial inner membrane. UCP-1 allows protons to enter the mitochondrial matrix at a lower energy with the proton leak [48]. Fucoxanthin induces the expression of UCP1 in abdominal white adipose tissue (WAT) [51]. This phenomenon is also known as browning, where WAT changes in phenotype into brown adipose tissue (BAT) [52]. BAT plays a role in dissipating energy through heat production, unlike WAT, which stores excess energy as triglycerides [52,53,54]. As the browning process occurs, energy expenditure in WAT is upregulated [55]. Through this process, the amount of WAT is reduced, which means fat accumulation is also reduced. This mechanism helps to treat NAFLD.

Fucoxanthin induces loss of WAT and alleviates hyperglycemia in an obese–diabetic KK-A(y) mouse model [56]. The same effect was also shown in high-fat-diet (HFD)-induced obese mice, as well as hyperinsulinemia and hyperleptinemia effects [57]. The decrease in WAT is consistently followed by an increase in BAT weight [58,59,60]. Further, the expression of UCP-1 is also increased in the WAT of KK-A(y) mice fed with fucoxanthin [61]. This anti-obesity activity of fucoxanthin was also examined in a human study, where according to Mikami (2017), fucoxanthin reduced HbA1c levels in subjects with G/G alleles of the UCP1 gene compared to those with the A/A and A/G alleles (thrifty allele of UCP1-3826A/G) in a human study consisting of 60 normal weight and obese Japanese adults with a BMI over 22 [62]. By lowering the level of HbA1c, fucoxanthin supplementation succeeded in lowering blood sugar levels, with no significant effect on visceral fat.

UCP1 is related to other mitochondrial metabolite transporters, such as the adenine nucleotide translocator, a proton channel in the mitochondrial inner membrane that permits the translocation of protons from the mitochondrial intermembrane space to the mitochondrial matrix. Other than the upregulation of UCP1, the expression of the β3-adrenergic receptor (β3Ad) and peroxisome proliferator-activated receptor gamma coactivator 1 (PGC-1) is also upregulated in WAT [57,63]. Activation of PGC-1 induces mitochondrial biogenesis [62]. Fucoxanthin is presumed to induce mitochondrial biogenesis.

### 3.2. Fucoxanthin Alters Lipid Metabolism

Lipid metabolism can be elucidated in two ways, lipolysis and lipogenesis. Both work in opposing manners, where lipogenesis synthesizes fat and lipolysis breaks down fat. Fat accumulation, which leads to obesity, results when an imbalance in lipogenesis and lipolysis occurs [64]. One of NAFLD’s characteristics is an alteration in the lipid metabolism that is also observed in atherogenic dyslipidemia [65]. An increase in de novo lipogenesis is considered the major alteration of lipid metabolism in NAFLD [66]. As a result, hepatic steatosis, in which the intrahepatic fat of more than 5% of the liver’s weight is accumulated, occurs through an increase in liver fatty acids and downregulation of β-oxidation [67].

**Figure 4:**
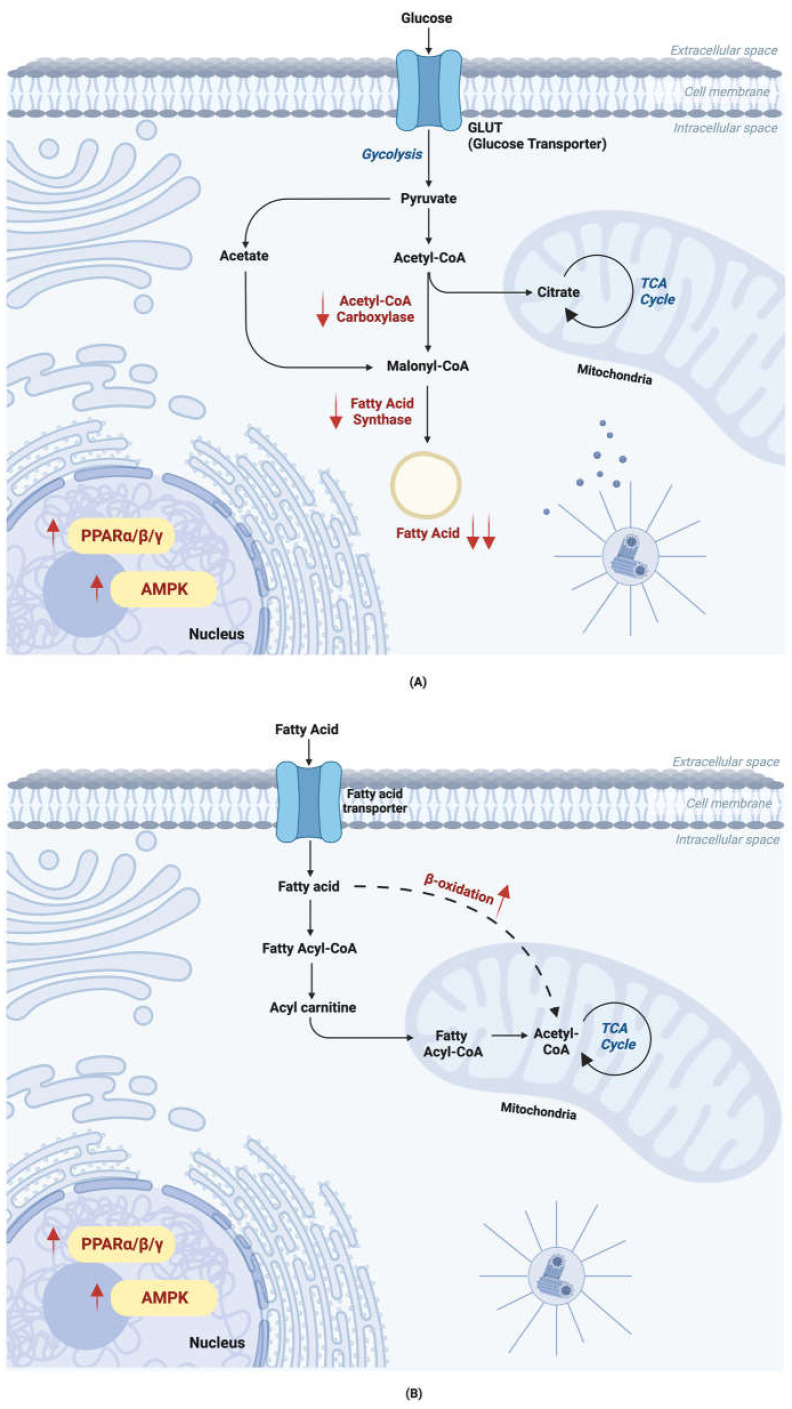
**Fucoxanthin alters lipid metabolism.** (**A**) Lipogenesis. Fucoxanthin downregulates acetyl CoA carboxylase and fatty acid synthase in lipogenesis, decreasing the lipid content in the liver. (**B**) Lipolysis. Fucoxanthin promotes β-oxidation, hence resulting in upregulated lipolysis in the liver. The downward arrow indicates downregulation and the upward arrow indicates upregulation.

Other than inducing thermogenesis activity via UCP-1 activation, fucoxanthin also functions by altering lipid metabolism and absorption [68], as shown in Figure 4 by inhibiting lipogenesis while promoting lipolysis. This mechanism works against NAFLD-altered lipid metabolism. Fucoxanthin upregulates enzymes related to lipolysis while downregulating enzymes related to lipogenesis. In HFD-fed mice, fucoxanthin can decrease hepatic lipid and plasma triacylglycerol levels [7,69]. These effects were shown through the increase in undigested fecal lipids. Fucoxanthin also functions by reducing the activity of hepatic lipogenesis and upregulating the activity of fatty acid β-oxidation [7]. It upregulates other key proteins in lipid metabolism, such as AMP-activated protein kinase and acetyl-CoA carboxylase in epididymal adipose tissue. It also induces β3Ad [51], which upregulates lipolysis and thermogenesis [70].

The supplementation of fucoxanthin in an obese mouse model induced through HFD (20% fat) decreased the visceral fat pads without altering the food intake [69]. It also increased adiponectin levels and, on the other hand, decreased leptin levels in plasma. Both adiponectin and leptin are adipose-derived hormones that act as messengers to deliver signals from the adipose tissues to other tissues and organs [71]. Adiponectin has been reported to act as an insulin-sensitizing adipokine in heterozygous peroxisome proliferator-activated receptor (PPAR) β knockout mice, which protects it from HFD-induced obesity [72]. PPAR γ is also reported to be downregulated in 3T3-L1 adipocytes with fucoxanthin supplementation [8]. Fucoxanthin supplementation through *Nitzschia laevis* extract (NLE) has also been reported to decrease abdominal fat and hepatic steatosis in C57BL/6J mice and to avert the accumulation of lipids in HepG2 cells. It elevates mitochondrial activity, shown through the enhanced oxygen consumption rate and mitochondrial membrane potential, and phosphorylated acetyl-CoA carboxylase [67]. The phosphorylation of acetyl-CoA carboxylase inhibits lipogenesis in the hepatocytes of rats and prevents the accumulation of hepatic lipids [73].

Standardized fucoxanthin extract from *Phaeodactylum tricornutum* showed inhibitory activity toward lipogenesis in 3T3-L1 adipocytes by decreasing intracellular lipid contents without any cytotoxicity [8]. Another fucoxanthin extract from *Petalonia binghamiae* has been reported to suppress the accumulation of lipid droplets in the liver as well as alanine and aspartate transaminase serum levels. It functions by upregulating the adenosine monophosphate-activated protein kinase (AMPK) signaling pathway, targeting acetyl-CoA carboxylase in adipocytes as well as fatty acid β-oxidation [74]. The fatty acid synthase (FAS) protein, which is also included in the AMPK signaling pathway, is involved in lipogenesis and has also been reported to be downregulated in the livers of *db/db* diabetic mice [19,75]. Further, the expression of PPAR α, phosphorylated acetyl-CoA carboxylase, and carnitine palmitoyltransferase 1 was upregulated. Similarly, the fucoxanthin extracted from *Undaria pinnatifida* also exhibits an anti-obesity effect in the HFD mouse model, which was demonstrated by decreased visceral fat and hepatic lipid accumulation, as well as decreased adipocyte size [9,76].

Fucoxanthin as a medication for NAFLD has recently reached clinical trials. The administration of fucoxanthin combined with fucoidan for 6 months in 21 patients with NAFLD attenuated hepatic lipotoxicity. The fucoxanthin–fucoidan treatment succeeded in lowering triglyceride, total cholesterol, alanine transaminase (ALT), as well as aspartate aminotransferase (AST) in a high fat diet mouse model administered 200 or 400 mg/kg bw fucoidan–fucoxanthin. It also significantly reduced the NAFLD-induced inflammatory cytokines IL-6 and IFN-γ. Leptin and adiponectin were also altered favorably to fight against NAFLD [77]. However, fucoxanthin at low concentrations, 0.015% and 0.03% *w/w*, did not effectively reduce triglycerides and total cholesterol in the high fat diet mouse model [78]. Another study with the same high fat diet mouse model accompanied by fucoxanthin supplementation at 0.05% or 0.2% *w/w* successfully decreased triglyceride and cholesterol levels [76]. Hence, the effects of fucoxanthin on altering lipid metabolism, specifically in the high fat diet mouse model, are dependent on dose and feed composition. Further studies should be conducted in order to determine the minimum effective dose of fucoxanthin in this model.

### 3.3. Anti-Inflammatory Activity of Fucoxanthin

NAFLD has a broad spectrum of characteristics, including inflammation. Moreover, obesity is also categorized as low-level inflammation marked with abnormally produced inflammatory adipocytokines [79]. Inflammation in NAFLD is triggered by an excessive accumulation of lipids, which activates hepatic fibrosis [80]. NAFLD progression is affected by the balance between pro- and anti-inflammatory stimuli. Once inflammatory cells in the liver are activated, steatosis occurs, and inflammation develops. An increase in fatty acids in the liver will also increase systemic inflammation, which is commonly observed in NAFLD patients [81].

In NAFLD, chronic inflammation has also been proposed to be promoted by insulin resistance [82]. The inflammation in NAFLD is characterized by the upregulation of several cytokines, including IL-6, IL-1β, and TNF-α. This fact was proven by an in vivo study with the NAFLD mouse model, which resulted in elevated TNF-α levels in the liver [83]. Another in vivo study using the NAFLD-HFD mouse model exhibited similar results, where AST, ALP, leptin, cholesterol, triglyceride, TNF-α, and TGF-β levels were significantly increased [84]. It can be concluded that pro-inflammatory cytokines are upregulated in NAFLD.

Fucoxanthin exhibits anti-inflammatory activity, as proven by the various studies indicated in Table 1. Fucoxanthin can alter PPAR signaling pathways. It is well known that the PPAR signaling pathways are related not only to lipid metabolism but also inflammation. They control inflammation and the immune response by regulating macrophages [85]. Therefore, modulation of the PPAR signaling pathway for anti-obesity effects will simultaneously exhibit an anti-inflammatory effect. This theory was proven by several studies focusing on the anti-inflammatory effect of fucoxanthin. Fucoxanthin supplementation successfully suppressed IL-1β, TNF-α, COX-2, and iNOS in an HFD mouse model [79]. It also suppressed pro-inflammatory factors such as NO, PGE_2_, IL-1β, TNF-α, and IL-6 in RAW 264.7 cells via the NF-κB and MAPK signaling pathways [86]. Fucoxanthin supplementation in a diabetic/obese KK-A^y^ mouse model succeeded in downregulating the expression of inflammatory cytokines such as TNF-α, IL-6, and monocyte chemoattractant protein-1 (Mcp-1). Fucoxanthin also inhibited macrophage infiltration into the white adipose tissues of the mice [43].

An in vitro study using Raw264.7 macrophages successfully showed the downregulation of IL-10, IL-6, iNOS, COX-2, and NF-κB signals [87]. In vivo studies using the LPS-induced sepsis mouse model also exhibited the same results, where inflammatory cytokines such as IL-6, IL-1β, and TNF-α were downregulated after fucoxanthin supplementation. Fucoxanthin is presumed to inhibit the phosphorylation of the NF-κB signaling pathway at the cellular level and block nuclear translocation [88]. Fucoxanthin also downregulated iNOS and COX-2 expression in a carrageenan-induced paw edema mouse experiment through the MAPK, Akt, and NF-κB signaling pathways [89]. Other interleukins, such as IL-4, IL-5, IL-8, and IL-13, were also reported to be downregulated in asthmatic mouse models [90].

**Table 1:** Inflammatory cytokines altered after fucoxanthin supplementation.

Inflammatory Cytokines	Experimental Model	Dose	References
↓ IL-1β	High-fat-diet-induced obese mice	0.2, 0.4, or 0.6%	[79]
	RAW 264.7 macrophages	*I. okamurae*-extracted fucoxanthin; 12.5, 25, or 50 μM	[86]
	RAW 264.7 macrophages	5, 10, or 20 μM	[87]
↓ IL-4 ↓ IL-5	OVA-stimulated (OVA) mice	10 or 30 μM	[90]
	Bronchoalveolar lavage fluid (BALF) from asthmatic mice	10 mg/kg or 30 mg/kg	
	OVA-stimulated (OVA) mice	10 or 30 μM	
↓ IL-6	RAW 264.7 macrophages cells	*I. okamurae*-extracted fucoxanthin; 12.5, 25, or 50 μM	[86]
	BEAS-2B cells	3, 10, or 30 μM	[90]
	Bronchoalveolar lavage fluid (BALF) from asthmatic mice	10 mg/kg or 30 mg/kg	
	RAW 264.7 macrophages	5, 10, or 20 μM	[87]
↓ IL-8	BEAS-2B cells	3, 10, or 30 μM	[90]
	Bronchoalveolar lavage fluid (BALF) from asthmatic mice	10 mg/kg or 30 mg/kg	
↓ IL-10	RAW 264.7 macrophages	5, 10, or 20 μM	[87]
↓ IL-13	Bronchoalveolar lavage fluid (BALF) from asthmatic mice	10 mg/kg or 30 mg/kg	[90]
	OVA-stimulated (OVA) mice	10 or 30 μM	
↓ TNF-α	High-fat-diet-induced obese mice	0.2, 0.4, 0.6%	[79]
	RAW 264.7 macrophages cells	*I. okamurae*-extracted fucoxanthin; 12.5, 25, or 50 μM	[86]
	Bronchoalveolar lavage fluid (BALF) from asthmatic mice	10 mg/kg or 30 mg/kg	[90]
	OVA-stimulated (OVA) mice	10 or 30 μM	
↓ COX-2	High-fat-diet-induced obese mice	0.2, 0.4, or 0.6%	[79]
	RAW 264.7 macrophages	5, 10, or 20 μM	[87]
↓ iNOS			

↑—upregulated; ↓—downregulated.

### 3.4. Anti-Oxidant Activity of Fucoxanthin against NAFLD

Inflammation is correlated with reactive oxygen species (ROS), where increased ROS promote inflammation [91,92]. The increase in oxidative stress caused by excessive ROS production is one of the causes of NAFLD. Excess production of ROS has also been shown in patients with type 2 diabetes, insulin resistance, obesity, NASH, and NAFLD [81]. ROS are highly reactive and unstable, so they often lead to an imbalance in the bioavailability of the cellular anti-oxidant system [93]. When that occurs, it disrupts intracellular metabolism and modifies the functional role of cellular enzymes, structural proteins, and even cell membranes [94].

Oxidative stress may also occur as a form of lipid-induced stress. Lipid accumulation in the liver may induce oxidative stress by altering mitochondrial activity and function and disrupting the anti-oxidant system. ROS are involved in the mitochondrial respiratory chain. In response to fatty acid oxidation disruption, which takes place in mitochondria, the production of ROS may be increased. This may also lead to cell apoptosis; hence, oxidative stress may be a factor in steatosis inflammatory progression [81]. In NAFLD, the excess ROS affect lipid peroxidation and impair mitochondrial and peroxisomal oxidation of fatty acids, resulting in the release of inflammatory cytokines [95], as well as the production of pro-inflammatory cytokines via MAPK phosphorylation [91].

Although fucoxanthin lacks pro-vitamin A activity, it has unique properties as a carotenoid that exhibits anti-oxidant activity [96]. Through its anti-oxidant activity, it exhibits anti-inflammatory effects related to obesity. ROS formation was reduced when PC12 cells were treated with fucoxanthin [79]. Another study using 3T3-L1 cells showed a decrease in enzymes related to the production of ROS, such as nicotinamide adenine dinucleotide phosphate (NADPH) oxidase 4 (NOX4), the NADPH-generating enzyme, and glucose-6-phosphate dehydrogenase after fucoxanthin treatment [97]. Fucoxanthin also exhibits free radical scavenging activity, as shown by several studies. Fucoxanthin treatment succeeded in reducing doxorubicin-induced ROS compared to primary cardiomyocytes treated with doxorubicin alone, indicating that the anti-oxidant effect of fucoxanthin exerts a cardioprotective effect [98]. Fucoxanthin supplementation in a diabetic/obese KK-A^y^ mouse model reduced oxidative stress by alleviating lipolysis and downregulating lipogenesis through the sirtuin1/adenosine monophosphate-activated protein kinase (Sirt1/AMPK) pathway in lipid-loaded hepatocytes [43].

## 4. Preventive Effect of Fucoxanthin on NASH Development

NAFLD is a progressive disease that may develop into non-alcoholic steatohepatitis (NASH), fibrosis, cirrhosis, and hepatocellular carcinoma. When it develops into NASH, fibrosis is one of its characteristics and the main cause of mortality [99]. Fucoxanthin not only exhibits a beneficial effect on NAFLD but also has a preventive effect on averting its development into NASH through fibrosis. The regulation of chemokine production suppresses hepatic inflammation and infiltration of immune cells, which is the leading cause of NASH development.

Fucoxanthin is reported to downregulate the hepatic mRNA expression of Tgfβ1, collagen type I alpha 1 chain (Col1α1), and Timp1 in the CDAHFD-fed mouse model [42]. Tgfβ1, transforming growth factor beta 1, is a pro-fibrogenic cytokine that expresses α-smooth muscle actin (αSMA) and promotes extracellular matrix (ECM) production [100]. The suppression of Tgfβ1 inhibits the production of ECM, which prevents fibrosis development. Tgfβ1-induced fibrosis is strongly correlated with matrix metalloproteinase-1 (TIMP-1) expression [101]. Timp1 plays a role in ECM degradation, where the administration of an anti-TIMP-1 antibody ameliorated fibrosis in mice [42].

Fucoxanthin was also reported to downregulate TGFβ1-induced mRNA levels of fibrogenic genes in LX-2 cells. It alleviated the phosphorylation of SMA- and MAD-related protein (SMAD3), which inhibits fibrosis. It exhibited a synergistic effect with SIS3 (an inhibitor of SMAD3) in suppressing fibrogenic gene expression. A similar result has also been reported in hepatic stellate cells. Its anti-fibrogenic activity is further explained through the repression of the NADPH oxidase 4 (NOX4) mRNA levels, which prevented the accumulation of ROS by TGFβ1 [102]. Hence, fucoxanthin exhibits anti-fibrogenic activity that prevents NASH development.

## 5. Signaling Pathways Altered by Fucoxanthin

**Figure 5:**
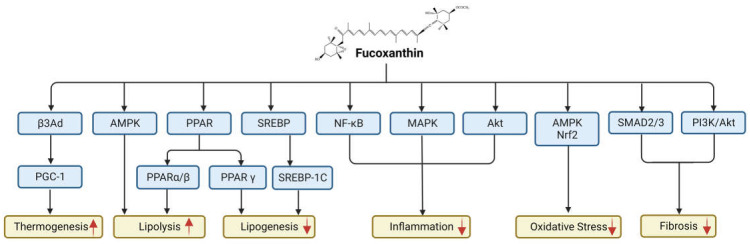
**Signaling pathways altered by fucoxanthin.** Fucoxanthin alters β-3-adrenergic receptor (β3Ad), adenosine monophosphate-activated protein kinase (AMPK), peroxisome proliferator-activated receptor (PPAR), sterol regulatory element binding protein (SREBP), nuclear factor-κB (NF-κB), mitogen-activated protein kinase (MAPK), and AKT pathways.

Fucoxanthin alters several pathways related to NAFLD, as shown in Figure 5 and Table 2. β3Ad is a metabolic receptor in adipose tissues. The upregulation of β3Ad has been closely related to thermogenesis [103]. A PPAR γ coactivator, PGC-1, is a transcription cofactor that plays a role in regulating cell metabolism [104]. Both PGC-1 and β3Ad stimulate adaptive thermogenesis and mitochondrial biogenesis that favor anti-obesity activity against NAFLD.

The AMPK signaling pathway regulates several metabolic organs, such as the liver, skeletal muscle, pancreas, and adipose tissues. AMPK pathways regulate glucose transport and fatty acid oxidation in skeletal muscle. They upregulate fatty acid oxidation in the liver while decreasing cholesterol and triglyceride synthesis [112]. Several studies have shown that the activation of AMPK results in acetyl-CoA carboxylase suppression, hence blocking fatty acid synthase and decreasing hepatocytic lipid accumulation [43]. The activation of AMPK specific to the liver has been reported to reduce liver steatosis, inflammation, and fibrosis in NAFLD patients. Liver-specific activation of AMPK made mice resistant to weight gain and reduced the overall level of lipid accumulation [113]. Fucoxanthin upregulates AMPK, hence promoting fatty acid oxidation to protect against NAFLD.

Other transcription factors related to lipid metabolism, such as PPARs and sterol regulatory element binding protein (SREBP), are also altered. Similar to AMPK, SREBP regulates the expression of lipogenic enzymes, including fatty acid synthase, acetyl-CoA carboxylase, and 3-hydroxy-3-methylglutaryl-CoA reductase [114]. SREBP-1C is one of the major transcriptional factors involved in de novo lipid synthesis, which affects NAFLD through the nuclear transcription factor farnesoid X receptor (FXR) [41]. Along with stearoyl coenzyme-A desaturase 1 and fatty acid synthase, activation of SREBP-1C increases the rate of fatty acid synthesis. The overexpression of SREBP-1C results in the upregulation of lipogenesis; meanwhile, inactivation of the SREBP-1C gene can reduced triglyceride levels up to 50% in the *ob*/*ob* mouse model [115]. Fucoxanthin has been reported to downregulate SREBP-1C; hence, it is believed to reduce lipogenesis and is beneficial for treating NAFLD.

Meanwhile, PPARs have an important role in regulating glucose levels and homeostasis, as well as regulating cell proliferation, differentiation, and inflammation [116]. PPARs are sensors of fatty acids and have tissue-specific expression patterns. PPAR-α is mainly expressed in brown adipose tissues and the liver and regulates lipid metabolism. PPAR-α alteration may lead to hepatic steatosis. Meanwhile, PPAR-β regulates oxidative metabolism (β-oxidation of fatty acids). PPAR-γ is mainly expressed in adipose tissues and macrophages to regulate adipogenesis and storage of fatty acid as triacylglycerol [117]. The activation of PPAR-γ is closely related to obesity, excess nutrients, and the storage of fatty acids as lipids [118]. Fucoxanthin upregulates PPAR-α and PPAR-β while downregulating PPAR-γ [7,9,76]. It functions by upregulating fatty acid β-oxidation and downregulating lipid storage as triacylglycerol in the liver. Fucoxanthin supplementation can alter the PPAR pathway in a favorable manner against NAFLD.

Fucoxanthin downregulated NF-κB, MAPK, and AKT signaling pathways in response to inflammation [86,89]. NF-κB is one of these inflammatory signaling pathways [119]. It plays a role in the homeostasis and expressing the immune response during the cell cycle [120]. Along with FOXP3, NF-κB regulates the secretion of inflammatory cytokines and chemokines. It is a major inducible transcription factor and primarily a cytoplasmic factor expressed in most types of cells [121]. Downregulation of this signaling pathway is related to the alleviation of liver inflammation and improvement in liver histopathology, as shown in rats with type 2 diabetes mellitus (T2DM) and NAFLD treated with liraglutide or hUC-MSCs [122]. Mice with NAFLD induced by a methionine–choline deficient diet (MCDD) showed that both the NF-κB and AKT signaling pathways were downregulated in response to lower levels of inflammation [123].

MAPK is another fundamental inflammation signaling pathway that drives expression of nuclear factor E2-related factor 2 (Nrf2) and NF-κB [124]. MAPK has been found to be responsible for lipid accumulation, inflammation, and ROS production in the HFD mouse model [125]. The suppression of this pathway reduces the inflammatory response in many diseases. The AKT pathway plays a role in cell metabolism, mainly in glucose metabolism. It is also closely associated with cancer and diabetes [126]. Alteration of this pathway is also related to obesity [127]. Fucoxanthin plays a valuable role in downregulating these three major inflammatory signaling pathways.

The anti-oxidant activity of fucoxanthin is closely related to the Nrf2 and AMPK signaling pathway. Fucoxanthin activates the Nrf2 and AMPK signaling pathway to reduce oxidative stress [109,110]. Activation of the AMPK pathway in the liver has been shown to improve the mitochondria’s ability to resist oxidative damage. Meanwhile, the anti-fibrogenic activity of fucoxanthin is mainly through the inhibition of TGF-β1. One of the TGF-β1-related signaling pathways is the SMAD signaling pathway. Fucoxanthin supplementation inhibited TGF-β1 and altered the TGF-β1-dependent SMAD, MAPK signaling pathways, and the phosphatidylinositol 3-kinase (PI3K)/Akt pathway [111]. A recent study using an HPF cell model showed that fucoxantin’s anti-fibrogenic inhibition of TGF-β1 is critically dependent on the SMAD2/3 signaling pathway.

## 6. Conclusions and Further Potential of Fucoxanthin against NAFLD

The biological activities of fucoxanthin against NAFLD hold promising prospects. However, there are limitations in commercialization due to the high cost of extraction with low yield, low bioavailability, and instability. Various delivery systems for fucoxanthin are currently being developed to increase its bioavailability. Encapsulated fucoxanthin has been proven to have a higher bioavailability in several recent studies [128]. Encapsulation has been attempted using hydroxypropyl-β-cyclodextrin, gum arabic, maltodextrin, gelatin, isolated pea protein, whey protein, zein mixed with caseinate, κ-carrageenan, and poly(d,l-lactic-co-glycolic acid) [129,130,131]. All encapsulation efficiencies were higher than 80%. In addition to encapsulation using a single material, the combination of a protein and polysaccharide is emerging as a major trend for complex delivery systems. Several complex encapsulations have been conducted using arabic/gelatin, whey protein isolate and Ca2+ cross-linked flaxseed gum, zein/chitosan, and nano-encapsulation using gliadin and chondroitin sulfate [132,133,134]. Protein–protein combinations such as lysozyme, protein–lipid combinations such as bovine serum albumin (BSA) and oleic acid, and polysaccharide–lipid combinations such as chitosan–bacuri butter and tucumã oil have also been reported [135,136,137].

Fucoxanthin functions against NAFLD through its thermogenic activity in mitochondrial homeostasis, altering lipid metabolism, its anti-inflammatory and anti-oxidant activities. The thermogenesis activity of fucoxanthin functions against NAFLD via UCP1 activation. Recently, UCP1 has been expected to form a complex with mitochondrial calcium uniporter (MCU) and essential MCU regulator (EMRE), named the thermoporter [40]. While UCP1 is associated with proton leakage, MCU and EMRE are associated with calcium uptake in the mitochondria membrane. As fucoxanthin is known for its thermogenic effect through UCP-1, if UCP-1 indeed forms a complex with EMRE/MCU, mitochondrial calcium uptake regulation may affect UCP-1 and its thermogenic activity. Thus, fucoxanthin’s thermogenic activity could be optimized by finding a compound that synergistically regulates calcium uptake. 

Lipid metabolism in the human body is actively altered by changes in diet. De novo lipogenesis plays a major role in NAFLD. It leads to hepatocytic accumulation of triglycerides [73]. Polyunsaturated fatty acid supplementation suppresses lipogenesis gene expression in the liver, including fatty acid synthase, spot14, and stearoyl-CoA desaturase [138]. Meanwhile, a high-carbohydrate diet stimulates lipogenesis in adipose tissues as well as the liver, as indicated by the elevated level of triglycerides [139]. This mechanism can be utilized to find a compound works synergistically with fucoxanthin that can help fight against NAFLD. The conversion step of acetyl-CoA to malonyl-CoA by acetyl-CoA carboxylase is the rate-limiting step in de novo lipogenesis, where malonyl-CoA also plays a role in regulating mitochondrial fat oxidation by inhibiting carnitine palmitoyltransferase I [73]. Soraphen A, a natural polyketide compound isolated from the bacterium *Sorangium cellulosum*, has been reported to inhibit acetyl-CoA carboxylase and to increase insulin sensitivity in an HFD-fed, insulin-resistant mouse model. Piperidinyl derivative CP-610431, spirocyclic spiropiperidine-derived compound, olumacostat glasaretil, aryl ether-derived analog, piperazine oxadiazole, and 1,4-disubstituted cyclohexane are also other reported acetyl-CoA carboxylase inhibitors [140]. Finding a synergistic inhibitor of acetyl-CoA carboxylase may help fucoxanthin in combating NAFLD.

Further studies on the role of fucoxanthin against NAFLD should also be performed at the level of RNA. Many RNA studies have been conducted regarding NAFLD because of its role in signaling pathways [141]. Non-coding RNA can effectively silence gene expression and alter signaling pathways. A long non-coding RNA highly upregulated in liver cancer (lncRNA HULC) has been found to inhibit the MAPK signaling pathway in an NAFLD mouse model [142]. Locked nucleic acid (LNA) was also found to inhibit lipogenesis and upregulate fatty acid oxidation in *db/db* mice [143]. A microRNA, miR-291b-3p, affects the AMPK signaling pathway by inhibiting fatty acid synthesis as well as de novo lipogenesis [144]. The microRNA miR-378 alters the AKT signaling pathway and reduces lipogenesis [145]. In another study of 16S rRNA, it was found that fucoxanthin altered high-fat-diet-induced gut microbiota dysbiosis by suppressing the growth of obesityinflammation-related *Lachnospiraceae* and *Erysipelotrichaceae* gut bacteria and inducing the growth of *Lactobacillus/Lactococcus*, *Bifidobacterium*, and butyrate-producing gut bacteria [146]. A similar study also showed that fucoxanthin altered the *Firmicutes*/*Bacteroidetes* ratio and the abundance of *S24-7* and *Akkermansia*, which attenuates obesity in a high fat diet mouse model [147]. Fucoxanthin regulates gut microbiota to treat NAFLD through its anti-obesity activity.

## Figures and Tables

**Table 2:** Transcriptional factors altered by fucoxanthin.

Transcriptional Factors	Experimental Model	Dose	Reference(s)
**Thermogenesis Related**			
↑ PGC-1	KK-A^y^ mice	*Undaria pinnatifida*-extracted fucoxanthin; 0.5% and 2% of control diet;	[51]
		0.2% fucoxanthin in control diet (AIN-93G)	[105]
	High-fat-diet-induced obese mice	0.69% *Undaria pinnatifida*-extracted fucoxanthin (2.9%)	[76]
↑ β3-adrenergic receptor (β3Ad)	KK-A^y^ mice	*Undaria pinnatifida*-extracted fucoxanthin; 0.5% and 2% of control diet;	[51]
	High-fat-diet-induced obese mice	1.06% or 2.22% in control diet (AIN-93G)	[57]
**Lipid Metabolism Related**			
↑ AMPK	High-fat-diet-induced obese mice	*Petalonia binghamiae*-extracted fucoxanthin; 150 mg/kg/day	[7]
↑ PPARα		*Undaria pinnatifida*-extracted fucoxanthin (2.9%); 0.69% *w/w*	[76]
↑ PPAR β		0.05% and 0.2% fucoxanthin, *w/w*	[9]
↓ PPAR γ	High-fat-diet-induced obese mice	0.05% and 0.2% fucoxanthin, *w/w*	[9]
		*Petalonia binghamiae*-extracted fucoxanthin; 150 mg/kg/day	[7]
		*Undaria pinnatifida*-extracted fucoxanthin (2.9%); 0.69% *w/w*	[76]
	3T3-L1	*Petalonia binghamiae*-extracted fucoxanthin; 10 μM	[106]
		fucoxanthin, fucoxanthinol, and amarouciaxanthin extracted from *U. pinnatifida*; 10 μM	[107]
↓ SREBP1c	High-fat-diet-induced obese mice	*Petalonia binghamiae*-extracted fucoxanthin; 150 mg/kg/day	[7]
**Inflammation Related**			
↓ NF-κB	RAW 264.7 macrophages	*I. okamurae*-extracted fucoxanthin; 12.5, 25, or 50 μM	[86]
	Carr-induced paw edema in ICR mice	*Undaria pinnatifida*-extracted fucoxanthin; 4 and 8 mg/kg	[89]
↓ MAPK	Macrophage RAW 264.7 cells	*I. okamurae*-extracted fucoxanthin; 12.5, 25, or 50 μM	[86]
	Carr-induced paw edema in ICR mice	*Undaria pinnatifida*-extracted fucoxanthin; 4 and 8 mg/kg	[89]
↓ Akt			
Anti-Oxidant Related			
↑ Nrf2	Alcoholic liver injury mouse model	10, 20, 40 mg/kg b.w.	[108]
	H9c2 cells	1 μM	[109]
↑ AMPK	HepC2 cells	*L. Japonica*-extracted fucoxanthin; 30 μg/mL	[110]
**Anti-Fibrogenic**			
↓ SMAD2/3	Human pulmonary fibroblasts (HPFs)	5, 10, 20 μM	[111]
↓ PI3K/Akt			
↓ MAPK			

↑—upregulated; ↓—downregulated.

## Data Availability

No new data were created.
